# Improved linking of motifs to their TFs using domain information

**DOI:** 10.1093/bioinformatics/btz855

**Published:** 2019-11-19

**Authors:** Nina Baumgarten, Florian Schmidt, Marcel H Schulz

**Affiliations:** 1 Institute for Cardiovascular Regeneration, Goethe University, Frankfurt am Main 60590, Germany; 2 German Center for Cardiovascular Regeneration, Partner Site Rhein-Main, Frankfurt am Main 60590, Germany; 3 High-throughput Genomics & Systems Biology, Cluster of Excellence MMCI, Saarland University; 4 Research Group Computational Biology, Max Planck Institute for Informatics, Saarland Informatics Campus, Saarbrücken 66123, Germany

## Abstract

**Motivation:**

A central aim of molecular biology is to identify mechanisms of transcriptional regulation. Transcription factors (TFs), which are DNA-binding proteins, are highly involved in these processes, thus a crucial information is to know where TFs interact with DNA and to be aware of the TFs’ DNA-binding motifs. For that reason, computational tools exist that link DNA-binding motifs to TFs either without sequence information or based on TF-associated sequences, e.g. identified via a chromatin immunoprecipitation followed by sequencing (ChIP-seq) experiment.

In this paper, we present *MASSIF*, a novel method to improve the performance of existing tools that link motifs to TFs relying on TF-associated sequences. *MASSIF* is based on the idea that a DNA-binding motif, which is correctly linked to a TF, should be assigned to a DNA-binding domain (DBD) similar to that of the mapped TF. Because DNA-binding motifs are in general not linked to DBDs, it is not possible to compare the DBD of a TF and the motif directly. Instead we created a DBD collection, which consist of TFs with a known DBD and an associated motif. This collection enables us to evaluate how likely it is that a linked motif and a TF of interest are associated to the same DBD. We named this similarity measure *domain score*, and represent it as a *P*-value. We developed two different ways to improve the performance of existing tools that link motifs to TFs based on TF-associated sequences: (i) using meta-analysis to combine *P*-values from one or several of these tools with the *P*-value of the domain score and (ii) filter unlikely motifs based on the domain score.

**Results:**

We demonstrate the functionality of *MASSIF* on several human ChIP-seq datasets, using either motifs from the HOCOMOCO database or *de novo* identified ones as input motifs. In addition, we show that both variants of our method improve the performance of tools that link motifs to TFs based on TF-associated sequences significantly independent of the considered DBD type.

**Availability and implementation:**

*MASSIF* is freely available online at https://github.com/SchulzLab/MASSIF.

**Supplementary information:**

Supplementary data are available at *Bioinformatics* online.

## 1 Introduction 

Transcription factors (TFs) are proteins that bind to DNA by recognizing specific DNA sequences with tertiary protein structures, so-called DNA-binding domains (DBDs) (Luscombe *et al.*, 2000). Thereby, TFs can regulate transcription by building complexes with other proteins, e.g. RNA polymerases (Reiter *et al.*, 2017). Recent studies suggest that TFs also directly influence chromatin state (Swinstead *et al.*, 2016). Besides, TFs are involved in many functional processes, e.g. maintaining the cell cycle, preserving and establishing specific cell types as well as inducing cell death (Vaquerizas *et al.*, 2009). Deregulation or mutations in TFs or mutations in TF-recognized sequences are the genetic trigger for many diseases (Deplancke *et al.*, 2016). Further details are elaborated in Lambert *et al.* (2018).

To understand the function of TFs, the composition of the sequences they bind to must be known. These sequences are called TF binding sites (TFBSs). Several experimental techniques are known to determine TFBSs *in vivo* or *in vitro* (Bulyk, 2007; Furey, 2012; Tuerk and Gold, 1990). To denote the sequence preference of a TF, all TFBS are summarized within TFBS patterns or motifs (in the following only motifs). These motifs are essential for computational inference of TFBSs and have been combined successfully with diverse epigenetic datasets to predict genome wide TF binding e.g. Pique-Regi *et al.* (2011) or Schmidt *et al.* (2018).

Currently, chromatin immunoprecipitation followed by sequencing (ChIP-seq) (Furey, 2012) is a popular technique to identify TFBS, since this *in vivo* method provides a genome wide and tissue-specific overview of TF binding. After peak calling the resulting DNA-sequences within the peaks are usually longer than the TFBS of the considered TF, with DNA-sequence lengths depending on the TF itself, the used peak caller and the quality of the data. Since TFBS are in general between 6 bp and 21 bp long, *de novo* motif discovery tools are commonly used to identify motifs enriched in these sequences (reviewed in Tompa *et al.*, 2005 or Tran and Huang, 2014). The result of a *de novo* motif discovery tool is a list of motifs, which were significantly enriched in the considered sequences. Not only the true motif of the TF can be detected, but also motifs of co-factors of the TF of interest, as well as repetitive sequences. Alternatively, methods like Clover (Chen *et al.*, 2004), PASTAA (Roider *et al.*, 2009), CentriMo (Bailey and Machanick, 2012), i-CisTarget (Potier *et al.*, 2015), REGGEA (Kehl *et al.*, 2018) or iRegulon (Janky *et al.*, 2014) make use of the increasing number of already known motifs linked to TFs to detect enriched motifs in the given sequences. The known motifs are usually taken from motif databases like JASPAR (Khan *et al.*, 2018), TRANSFAC (Matys *et al.*, 2006) or HOCOMOCO (Kulakovskiy *et al.*, 2018). Some of these methods can also handle *de novo* motifs as input; hence they are used as a follow-up analysis to eliminate repetitive motifs from the *de novo* motif discovery algorithm or to improve their ordering.

A closely related field of research tries to identify motifs of TFs independently of any associated DNA-sequences. One of the first studies (Tan *et al.*, 2005) that linked TFs to their motifs only used the information of the TFs derived from their amino acid sequences and thus also included the DBDs of the TFs. The motivation of this approach is that TFs associated to the same DBD are in general more similar to each other in terms of amino acid sequence and therefore tend to bind to similar motifs. Tan *et al.* (2005) used a probabilistic framework that included DBD similarity of motifs and comparative information for prediction in *Escherichia* *coli*. Later, it was shown, using a support vector regression model, that for some DBDs useful features from the protein sequence can be derived to predict the binding motif of a TF (Schröder *et al.*, 2010). However, the recent study by Zamanighomi *et al.* (2017) showed that using only features derived from the DBDs yield a high number of false positives (FPs). To overcome this problem, they combined the DBD-based information with a probabilistic model of motifs hits using epigenetic data.

In conclusion, the information derived from DBDs was successfully used in studies that link TFs to their motifs often independently from any TF-associated sequences. On the other hand, if tools are used that search for enriched motifs in TF-associated sequences, we recognized that these make no use of the powerful DBD information. Here, we introduce a method called *MASSIF*—motif association with domain information—that extends existing tools that link motifs to TFs and improves their performance using DBD information, utilizing a statistic comparable to Tan *et al.* (2005). We demonstrate that well-known and commonly used tools show significantly improved performance on real ChIP-seq data when combined with *MASSIF*.

## 2 Materials and methods

### 2.1 Overview

**Fig. 1.**
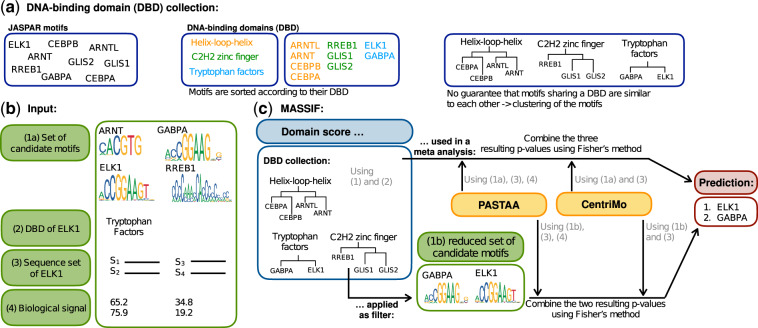
An overview of the DBD collection and *MASSIF* is shown. (**a**) Depicts the different steps of how the DBD collection is constructed. (**b**) Illustrates the necessary input for *MASSIF*. (**c**) Outlines the workflow of the two variants of *MASSIF*. The upper part of the figure displays the variant where the domain score is used in a meta-analysis. The lower one shows how to apply the domain score as a filter

Our approach is based on the idea that a motif linked to a TF of interest is more likely to be correct if the TF and the motif are associated to the same DBD. For TFs, the DBD is usually known, but motifs are in general not associated to DBDs. For that reason, it is not possible to compare the DBD of a TF and the linked motif directly. To enable the comparison, we constructed a DBD collection consisting of TFs with known DBDs and linked motifs. We use the DBD collection to compare the linked motif to all motifs in the collection which are associated to the DBD of the TF of interest. This results in a similarity measure, called domain score, which we represent as a *P*-value. We use the domain score in two different ways: (i) Using Fisher’s method as a meta-analysis (Fisher, 1934) to combine the domain score with the results of existing tools. (ii) Applying the domain score as a filter to reduce the motif set before applying an existing tool. An overview is shown in Figure 1.

### 2.2 Similarity measurement and clustering of position frequency matrices

During the construction of the DBD collection, we use a cluster algorithm for Position Frequency Matrices (PFMs) developed by Pape *et al.* (2008). Also, our domain score is based on the similarity measurement *S*^max^ defined in their work. Pape *et al.* developed a software package, called *Mosta*, which conducts motif similarity computation and motif clustering.

Two PFMs, *X* and *Y* are assumed to be similar if they describe a similar binding site, or to put it in another way, if they have a high number of overlapping hits in a random sequence. Therefore, *Mostas’* similarity concept between two PFMs is based on an overlap probability $\gamma_{X,Y}(k)$ at position *k* of *X* and *Y* as well as on the probabilities of independent hits for *X* and *Y* at this position *k*, denoted by *α_X_* and *α_Y._* The overlap probability $\gamma_{X,Y}(k)$ sums the probability for all possible words x∈X and y∈Y to overlap at a position *k*. In addition, *α_X_* is the probability that the words x∈X occur in a background model, equally for *Y*. Applying the logarithm to the ratio of the overlap probability and the product of the probabilities of independent hits for *X* and *Y*, gives the similarity $S_{X,Y}$:

(1) $$S_{X,Y}=\text{log}\;\left(\frac{\gamma_{X,Y}(k)}{\alpha_{X}\cdot \alpha_{Y}}\right).$$

The ratio describes the probability to observe two hits assuming the motifs *X* and *Y* represent a similar binding site, normalized by the probability to observe hits for *X* and *Y* assuming they do not describe a similar binding site. *MASSIF* applies a concept, also provided by *Mosta* called *S*^max^ which is based on Equation (1). *S*^max^ is a maximization over all possible *k’*s and it also considers the reverse complement of the motifs.

Based on the definition of the similarity between two PFMs, *Mosta* determine clusters in a set of motifs. Each resulting cluster contains a set of motifs which is represented by a consensus motif. Initially, all considered motifs are interpreted as a separate cluster containing one motif which is also the consensus motif at the same time. Then in a greedy fashion, clusters are merged using *S*^max^ as a similarity measure. The procedure stops if the motifs in a cluster are not similar enough to the cluster consensus motif. The algorithm terminates if all pairs of consensus motifs are considered at least once or if the similarity of the remaining motif pairs is too low.

### 2.3 DBD collection

To build the DBD collection, we used information from the motif database JASPAR (version from 2016). JASPAR contains known motifs linked to TFs. Each TF of the database is associated to a DBD, based on the TFClass system (Wingender *et al.*, 2013). In a first step, we separate the JASPAR TFs and hence, indirectly the motifs, according to their DBDs. The considered motifs belong to 30 different DBDs (listed in Supplementary Table S1).

Since the JASPAR database contains 515 motifs, several motifs are assigned to the same DBD. However, there is no guarantee that motifs associated to the same DBD are similar to each other. The domain score determines a similarity between a candidate motif and the motifs within a DBD, it is important to represent the diversity of motifs for a DBD as accurately as possible to avoid FPs as well as false negatives (FNs). The idea is to cluster motifs associated to the same DBD annotation into distinct groups, so that the motifs within each group are more similar to each other than to the motifs outside this group. Then we can compare the candidate motif to the different groups within a DBD to determine the domain score. Therefore, we cluster the corresponding PFMs of the motifs within a DBD using the cluster algorithm provided by *Mosta*. We get for each DBD a set of clusters *D_l_*, where l∈{1,…,30} since we observed 30 DBDs. Then D is defined as a the set of all DBDs $D=\{D_{1},\ldots ,D_{30}\}$. Each set of clusters *D_l_* consist of a varying number *n* of clusters $c_{l}^{k}$ with k∈{1,…,n} depending on how similar the motifs within the DBD are. Thus, we can write $D_{l}=\{c_{l}^{1},\ldots ,c_{l}^{n}\}$. In addition, the motifs within a cluster $c_{l}^{k}$ are represented by a consensus motif $M_{c_{l}^{k}}$. To sum up, the DBD collection contains 515 JASPAR motifs, which belong to 30 DBDs with an average number of 10 clusters per DBD (for detailed information, see Supplementary Section S4).

### 2.4 Domain score

To calculate the domain score between a motif and a TF, we need to be aware of the DBD of the TF. In general, for most of the TFs, the DBD is known and can be looked up, for instance in UniProt (The UniProt Consortium, 2017). Otherwise, if the DNA- or protein sequence is known, the DBD can be predicted using tools like SMART (Letunic *et al.*, 2015) or UniPROBE (Hume *et al.*, 2015).

Knowing the DBD of a TF, we can use the DBD collection to look up the consensus motifs of the set of clusters *C_l_* associated to this DBD. Based on that, the domain score is computed yielding for each candidate motif a score indicating how similar this candidate motif is to the most similar consensus motif of the DBD of the current TF. In more detail, we calculate the similarities *S*^max^ between the PFM *P* of the candidate motif and all consensus motifs $M_{c_{l}^{k}}$ of the DBD of the current TF. So, the set of similarities can be computed as following:

(2) $$\text{sim}(P,D_{l})=\{S^{\text{max}}(P,M_{c_{l}^{k}})\;|\;c_{l}^{k}\in C_{l}\}.$$

Among all calculated similarities, we pick the highest one, since the maximum similarity is achieved for the consensus motif that is most similar to the candidate motif. The consensus motifs of a DBD might be different to each other, thus the maximization is important. Since the similarity value also depends on the motif itself, we divide the maximal similarity by the sum of all maximal similarities over all DBDs to normalize for this effect. So, we get the following formula for the domain score *I_D_*:

(3) $$I_{D}(P,D_{l})=\frac{\max\;\text{sim}\;(P,D_{l})}{\sum_{m=1}^{\left|D\right|}\max\;\text{sim}\;(P,D_{m})}.$$

**Fig. 2.**
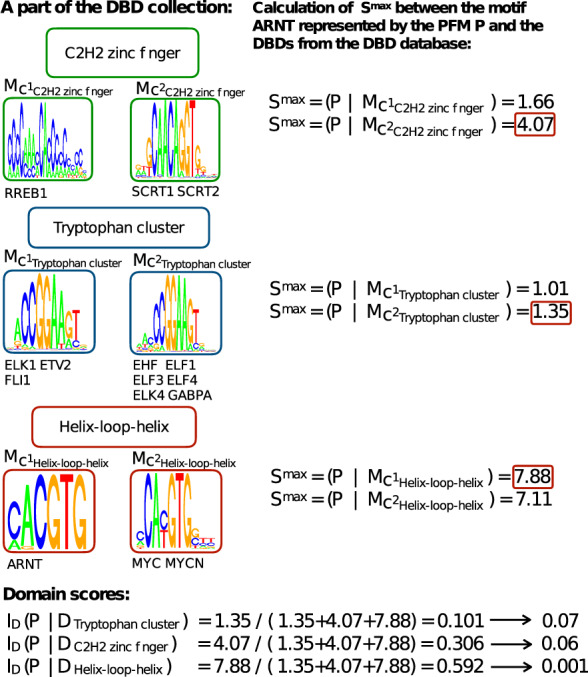
Example how to calculate the domain score on an excerpt of the DBD collection (left). For each DBD the corresponding consensus motifs are shown and the TFs within this cluster listed below. (Right) The similarities *S*^max^ between the PFM *P* (here for TF ARNT) and the consensus motifs of all DBDs are computed. (Bottom) The calculated domain scores and the corresponding *P*-values assuming the candidate TF has the DBD ‘Tryptophan cluster’, ‘C2H2 zinc finger’ or ‘Helix-loop-helix’ are shown

The higher the similarity the more likely the candidate motif has the same DBD as the current TF. Figure 2 illustrates an example how to calculate the domain score. To enable a better interpretation of the domain scores as well as to give us the possibility to use them in a statistical test, the domain scores are represented as *P*-values:

(4) $$P-\text{value}:=\mathrm{Pr}(I_{D}(P,D_{l})\geq x|H_{0}),$$

where *x* is an observed value of the domain score and the null hypothesis *H*_0_ is defined as: ‘the DBD of the current TF and the DBD associated to the motif are not the same’. Since we have no analytical description of *H*_0_, we approximate it by using Monte Carlo sampling. By randomly sampling 100 000 PFMs (average entropy ≥0.6, length between 6 and 21) we obtain a set of random PFMs *R*. For further information about the PFM sampling, see Supplementary Section S6. We calculate for each random motif r∈R the domain score *x* for a given DBD *D_l_* and we estimate a *P*-value for this score as follows:

(5) $$p(x,D_{l})=\frac{\left|\{r|I_{D}(r,D_{l})\geq x,r\in R\}\right|}{\left|R\right|}.$$

Basically, we count how often a random motif has an observed domain score for a given DBD that is higher than the score *x* and divide it by the total number of motifs in *R*.

The domain score can either be used in a meta-analysis or as a motif filter. In the following, both variants are explained in more detail.

#### 2.4.1 Domain scores used in a meta-analysis

Tools that link motifs to TFs relying on TF-associated sequences usually return a list of motifs describing the likelihood that the motifs are over-represented in the set of input sequences. For each motif a *P*-value is given that is used to compare the motifs with each other. For each motif within this list, we determined the domain score *I_D_* [Equation (3)] and the corresponding *P*-value as explained in Equation (5). A meta-analysis is performed by using Fisher’s method to combine the *P*-values of the used tools and the domain score:

(6) $$X(p_{1},\ldots ,p_{m})=-2\cdot \sum_{i=1}^{m}\;\text{log}\;(p_{i}),$$

where *p_i_* represents the *P*-value of the *i*th method, and *m* is the number of methods considered. Fisher’s method follows the $\chi^{2}$ distribution with 2*k* degrees of freedom (Fisher, 1934), hence we can obtain the corresponding *P*-value by computing $1-F_{2k}(x)$, where $F_{2k}(x)$ is the cumulative distribution function of the $\chi^{2}$ distribution.

#### 2.4.2 Domain score used as a motif filter

The idea of the filtering is to reasonably reduce the set of input motifs for a tool that links motifs to a TF depending on TF-associated sequences, since we observed that the performance is usually better if we consider a smaller number of input motifs (data not shown). Therefore, we choose a *P*-value *π*, such that we can decide if the *P*-value of a domain score is significant or not. If a *P*-value of the domain score is higher than the corresponding *π*, we assume that it is unlikely that the TF and the motif are associated to the same DBD and exclude the motif from the set of input motifs. On the other hand, if the *P*-value of the domain score is equal to or smaller than *π*, we keep the motif. Finally, we apply the considered tools on the reduced motif set and, when we use more than one tool, we combine the results with Fisher’s method.

### 2.5 ChIP-seq data preparation

We downloaded ChIP-seq data for DNA binding proteins for Homo sapiens from ENCODE (The ENCODE Project Consortium, 2012) (ENCODE accession numbers in Supplementary Table S2). The dataset includes sequence sets for 102 TFs assayed in K562. Here, we use *optimal IDR threshold peaks* peak calls in narrow bed format computed with the *uniform ENCODE processing pipeline* and version *GRCh38* of the human reference genome. Using these publicly accessible, and transparently processed files allows for easy reproducibility of our results. In case that more than one peak file is available, we randomly choose one. Next, we use the BEDTools (Quinlan and Hall, 2010) *getfasta* command to extract the genomic sequences corresponding to the ChIP-seq peaks. In the header of the fasta file, we list the genomic location of the sequences, extended with the *signalValue* provided in the bed files. If a tool that links motifs to TFs based on TF-associated sequences, requires a biological information we use this *signalValue*. Sets of different sequence length are obtained from the middle of the peaks.

### 2.6 Evaluation of the results

To assess the performance of the tested methods, we determine for how many TFs a motif was linked correctly. As motif input set, we use (i) motifs from the HOCOMOCO database (401 motifs) or (ii) *de novo* motifs using GimmeMotifs (van Heeringen and Veenstra, 2011). To account for similarity between different HOCOMOCO motifs in the evaluation, we combine similar motifs by clustering, using *Mosta*. Linked motifs that belong to the same cluster as the true motif are counted as correctly linked. GimmeMotifs applies a clustering step to reduce the redundancy of the identified *de novo* motifs within their analysis, hence we do not cluster the motifs again for the evaluation. To determine which *de novo* motif is the correct one, we calculated the similarity for each of them to the known motif of the TF of the current ChIP-seq dataset. For this, we used the similarity function *sstat* from *Mosta*, which determines the similarity between two PFMs. The *de novo* motif that is most similar to the motif of the TF is assumed to be the correct one, after checking all motifs manually.

Further, we calculate Precision-Recall (PR) curves. *Recall* is defined as the number of correctly linked motifs (TP) divided by the number of FNs plus TP e.g. $\text{recall}:=\frac{\text{TP}}{\text{TP}+\text{FN}}$. Further, *precision* is specified as the number of TP divided by TP plus the FPs e.g. $\text{precision}:=\frac{\text{TP}}{\text{TP}+\text{FP}}$. Additionally, we determine for each method shown in the PR-Curves the area under the curve (AUC).

## 3 Results

In this study, we consider the following task: Given a TF, to which no motif is linked, and a set of sequences that are associated with the TF, e.g. identified via a ChIP-seq experiment, the aim is to identify the correct motif. To solve this, we developed a tool, called *MASSIF*, which improves the performance of existing tools that link motifs to TFs depending on TF-associated sequences by using the DBD of a TF to calculate a domain score. This score is based on the assumption that a motif which is correctly linked to a TF, should be assigned to a similar DBD than the TF. Since we do in general not know the DBDs of the motifs, we cannot directly compare the DBDs of the linked motif and the TF of interest. We construct a DBD collection, which consists of TFs with known motifs associated to a DBD from the JASPAR database. The DBD collection allows us to determine how likely it is that a linked motif and a TF of interest are associated to the same DBD. We compute the domain score between the linked motif and the set of motifs associated with the DBD of the TF, which we looked up in the DBD collection. The domain score can either be used in a meta-analysis or as a motif filter. An overview of the DBD collection and *MASSIF* is provided in Figure 1.

### 3.1 Analysis of domain score distributions

**Fig. 3.**
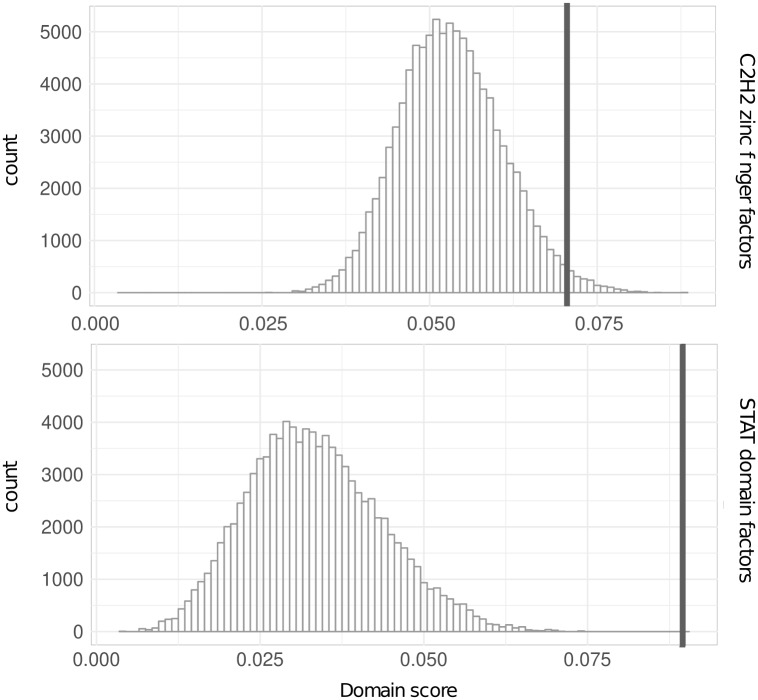
The distributions of the domain scores for two DBDs, namely ‘C2H2 zinc finger factors’ and ‘STAT domain factors’ are shown. The black vertical lines mark the smallest values of the domain score of all real motifs of the DBDs ‘C2H2 zinc finger factors’ and ‘STAT domain factors’

To decide if an observed domain score is significant or not, we calculate a domain score distribution for each DBD (Section 2.4). It is important to do this separately for each DBD, because the distributions of different DBDs heavily differ from each other (Fig. 3). Generally, the observed domain scores of DBDs that contain fewer motifs have a smaller mean than DBDs with a large number of motifs. An explanation for this effect is that the DBDs with a large number of motifs typically consist of more clusters than small ones. In addition, the bigger the clusters in a DBD the less specific are the consensus motifs, hence it is more likely that a randomly generated motif achieves a high similarity to the consensus motifs simply by chance.

### 3.2 Results on ENCODE data

*MASSIF* can only improve the performance of existing tools that link motifs to TFs relying on TF-associated sequences, hence we had to decide which of them we want to consider in our study. We choose CentriMo and PASTAA, because they are among the most used methods (McLeay and Bailey, 2010).

CentriMo (Bailey and Machanick, 2012) was designed for the analysis of ChIP-seq data and prioritizes motifs that are found in the middle of peak regions, by using a binomial test of motif occurrences in the center compared to border regions in the sequences.

PASTAA (Roider *et al.*, 2009) is a tool that uses not only the TF-associated sequences to link a motif but also biological information. In detail, PASTAA considers lists of ranked sequences based on TF binding signals for instance of a ChIP-seq experiment or tissue-specific gene expression data.

Both tools are user-friendly, easy to apply and have an acceptable run time even for large sequence sets. Especially runtime is important, because we validate *MASSIF* on a huge collection of ChIP-seq datasets. For an easy reproducibility of our results, the used commands for PASTAA and CentriMo are listed in Supplementary Section S1.

We consider 102 ChIP-seq datasets, where a linked motif for each chipped TF is known. To evaluate the performance of *MASSIF*, we use the sequences determined via the ChIP-seq experiment as input, and try to identify a motif for this TF. Whether we linked the correct motif or not, can be checked by comparing with the known true motif. To control if *MASSIF* improves the results of the considered approaches, that link motifs to TFs based on TF-associated sequences, we test multiple variations, for which we developed the naming scheme shown in Table 1. We first tested *MASSIF* with the known motifs of the HOCOMOCO database and than in a more difficult scenario, where we use *de novo* motifs as input motifs.

**Table 1.** Explanation and shortcuts of the different variants

	*C*	*P*	*CP*	*MCP*	*M_C_*	*M_P_*	*M_CP_*
Meta-analysis			*✓*	*✓*			*✓*
Domain score as filter					*✓*	*✓*	*✓*

*Notes*: ‘*C*’ and ‘*P*’ denote ‘CentriMo’ and ‘PASTAA’, respectively. ‘*M*’ followed by capital letter(s) denotes the use of the domain score in a meta-analysis and ‘*M*’ followed by subscript letter(s) the use of the domain score as filter.

#### 3.2.1 Results using HOCOMOCO motifs as input

**Fig. 4.**
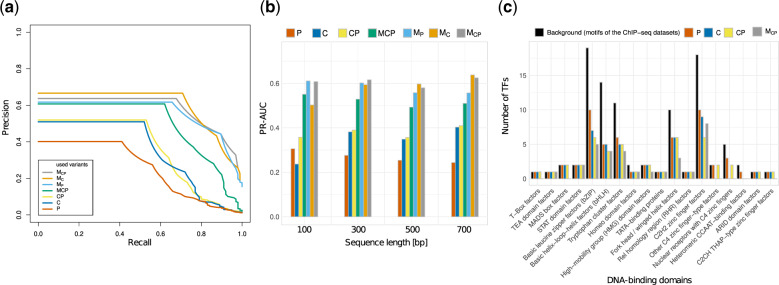
Results of the ENCODE dataset. (**a**) and (**b**) show the results of *MASSIF* using HOCOMOCO motifs for all downloaded ENCODE ChIP-seq dataset. (a) Outlines a PR curve for sequence length 500 bp, where the x-axis outlines the recall of the correctly linked to TFs and the y-axis the precision. (b) Bar plot illustrating the PR-AUC (y-axis) of the results for different sequence lengths (x-axis). (**c**) Analysis of the motifs incorrectly linked to a TF (y-axis) of some variants for sequence length 500 bp. The background represents the number of TFs per DBD of the input ChIP-seq datasets. The plot separates TFs by their DBDs (x-axis). For (a–c) the used variants are depicted with different colors

We run *MASSIF* for all variants shown in Table 1 on four sequence sets differing in the length (100 bp, 300 bp 500 bp and 700 bp), and evaluate the performance with the clustering evaluation as explained previously. The results are shown in Figure 4, where (a) shows exemplary the PR-Curve for all used variants for sequence length 500 bp and (b) the PR-AUCs for all variants for the different sequence lengths. To get a first clue how well PASTAA and CentriMo perform, we run them without any modifications. We observe that the PR-AUC of PASTAA is highest (≈0.310) for the shortest sequence length and drops for the longer ones. On the other hand, we see the opposite effect for the PR-AUC of CentriMo. The lowest PR-AUC (0.237) is observed for a sequence length of 100 bp, and the performance is improved for longer sequences e.g. a PR-AUC of 0.382 is achieved for a sequence length of 300 bp.

Next, we identified that the meta-analysis i.e. combining Pastaa and Centrimo within Fisher’s method, called *CP*, leads to an improvement in PR-AUC. The PR-AUC of this variant is similar compared to the PR-AUC of CentriMo (expect sequence length 100 bp), whereas an improvement to the PR-AUC of PASTAA is observed. In general, the performance of *CP* is more stable under varying sequence length compared to the results of CentriMo or PASTAA when used individually.

**Domain score used as** **meta-analysis**

To test if we can further improve the PR-AUC of *CP*, we add the *P*-values of the domain scores to the meta-analysis. We refer to this variant as *MCP*, which results in a clear improvement of the PR-AUC compared to *CP*. Especially, for the sequence length 100 bp the increase of correctly linked motifs is substantial. The average improvement of the PR-AUC of *MCP* over *CP* is around 0.142 for all sequence lengths.

**Domain score used as filtering**

Next, we test whether the *domain score* used as a filter leads to an improved PR-AUC. Therefore, we evaluate the results of PASTAA and CentriMo on the reduced motif set, termed *M_P_* and *M_C_*, separately. All motifs with π>0.001 are excluded. Interestingly, these variants improve many analysis. For sequence length 100 bp the PR-AUC of *M_P_* is 0.048 lower than the PR-AUC of *MCP*. On average over all sequence lengths the PR-AUC for *M_C_* compared to CentriMo improves by around 0.204 and *M_P_* compared to PASTAA by around 0.324. Still, both used tools show varying performance with the sequence length. For instance, the PR-AUC of *M_P_* drops by around 0.104 if we increase the sequence length from 100 bp to 700 bp.

Finally, we combine the variants *M_P_* and *M_C_* within the meta-analysis, and refer to it as *M_CP_*. Compared to *M_C_* the performance is similar except, for the shortest sequence length, where we observe an increase of the PR-AUC of 0.105. For *M_P_* the largest improvement is observed for longer sequences. Additionally, differences in the PR-AUC for varying sequence lengths are small compared to the meta-analysis or CentriMo and PASTAA. We observe the stable PR-AUC over all sequences for *M_CP_*, but for longer sequences the PR-AUC of *M_C_* is slightly higher. We conclude that adding the domain score leads to a substantial improvement for both methods with a slight improvement of the filter-based method over meta-analysis.

#### 3.2.2 Results using *de novo* motifs as input

As an alternative to the HOCOMOCO motifs, we investigate how *de novo* motifs affect the performance of *MASSIF*. To determine *de novo* motifs on the ENCODE ChIP-seq datasets, we applied a tool named *GimmeMotifs* (van Heeringen and Veenstra, 2011) on sequences of length 300 bp. We use this method for the following reasons: It identifies *de novo* motifs for ChIP-seq datasets in appropriate time and combines several *de novo* motif discovery tools within one method (used tools: MDmodule, MEME, Weeder, MotifSampler, trawler, Improbizer, BioProspector, Posmo, ChIPMunk, AMD, Homer and XXmotif). Additionally, *GimmeMotifs* clusters the resulting motifs to decrease the number of redundant ones. The algorithm was able to identify *de novo* motifs for 46 ChIP-seq datasets. We applied *MASSIF* on these datasets using the identified *de novo* motifs as input motifs.

**Fig. 5.**
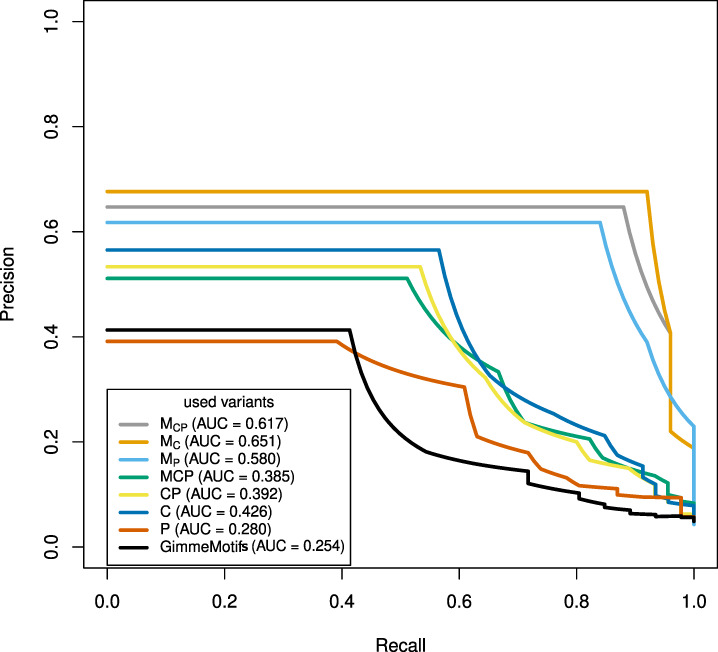
PR curve of the results of *MASSIF* with *de novo* identified motifs for 46 ENCODE ChIP-seq datasets (sequence length 300 bp). The x-axis outlines the recall of motifs correctly linked to TFs and the y-axis the precision

To evaluate how accurately the different variations perform, we followed the same strategy as before, and determined how many motifs are correctly linked to a TF. To determine which *de novo* motif is the correct one, we calculated the similarity for each motif to the known motif of the TF of the current ChIP-seq dataset as outlined in Section 2.6. Figure 5 shows the PR curve for the different variants. In addition to the variants used for *MASSIF* with HOCOMOCO motifs, the black curve represents the performance of *GimmeMotifs*, which sorts the *de novo* motifs according to their internal determined enrichment value.

We observe that in general the tendency of the results of *MASSIF* using *de novo* motifs as input are similar to the ones shown in Figure 4. The variants which use the domain score as a filter again perform best. Also the PR-AUC from CentriMo, PASTAA and *CP* are similar in comparison to *MASSIF* with HOCOMOCO motifs as input. Only the performance of *MCP* decreased and PASTAA performs better than GimmeMotifs.

In general, we noticed that some of the correctly linked *de novo* motifs are extremely similar to the true motif. Further, we observed that the filtering is able to remove *de novo* motifs that were highly different from the true one. For 12 out of 46 cases, the filtering rejected even all *de novo* motifs because of low similarity to any known motifs of the DBD of the current TF (π>0.001). Supplementary Section S4 outlines some examples of linked *de novo* motifs in comparison to the true one.

To conclude, the filtering can be applied as a quality control to eliminate *de novo* motifs that are found to be enriched in the sequences but most likely do not represent the true motif of the TF. Adding the domain score, especially as filter, leads to a considerable improvement of the performance of *MASSIF* with *de novo* identified input motifs.

### 3.3 Analysis of the motifs that are incorrectly linked to a TF

To get a better intuition, how the domain score influences the PR-AUC of the used tools that link motifs to TFs, we analyzed the motifs, which are incorrectly linked to a TF using the motifs from the HOCOMOCO database. In particular, we want to investigate for which DBDs using the domain score is helpful. In Figure 4c, the number of motifs that are incorrectly linked to a TF per DBD is shown. The black bars represent the background, which illustrates the number of TFs of the input ChIP-seq dataset per DBD. If we compare the number of motifs that are incorrectly linked to a TF of the different variants, we notice several interesting points.

First, in all cases *CP* is able to be at least as good as the performances of CentriMo or PASTAA. For four DBDs ‘Basic leucine zipper factors,’ ‘Basic helix-loop-helix factors’, ‘Tryptophan cluster factors’ and ‘C2H2 zinc finger factors’ the performance of *CP* achieves a better result than CentriMo or PASTAA alone. Interestingly, in two cases, namely ‘Other C4 zinc-type factors’ and ‘nuclear receptors with C4 zinc finger’, the performance of *CP* is poorer compared to the best used tool, that links motifs to TFs.

The performance for *M_CP_* is in most cases at least as good as the best considered variant or improves the performance. For the DBD, ‘C2H2 zinc finger factors’ *M_CP_* links two motifs incorrectly, which are correct linked from *CP*. In addition, we observe that *M_CP_* leads to an improvement of seven DBDs in such a way that all linked motifs are correctly associated, whereas without using the domain score at least PASTAA or CentriMo linked one motif incorrectly. Interestingly, five out of these seven DBDs contain only up to five motifs in the DBD collection, whereas the other two contain 11 and 36 motifs (data shown in the Supplementary Section S4). These findings suggest that for DBDs, which contain less motifs, the domain score strongly influences performance, which is consistent with the observation of Figure 3. There, we notice that it is easier for DBDs, which containing less motifs, to distinguish if a motif is correctly associated to a DBD or a FP one.

We conclude that, the reduced motif set which results from the filtering is much more specific, and contains fewer FPs for a TF associated to a DBD containing less motifs compared to a TF linked to a DBD, which contains more motifs. This can lead to better results for tools, that link motifs to TFs based on TF-associated sequences.

## 4 Discussion

In the present study, we analyzed how DBDs of TFs can be used to improve the performance of existing approaches, that link motifs to TFs relying on TF-associated sequences. Our tool *MASSIF* is based on the idea that a correctly linked motif and a TF of interest are associated with a similar DBD. However motifs are usually not linked to DBDs. As a solution, we construct a DBD collection that enables us to calculate how likely it is that a linked motif and the TF of interest are associated to the same DBD. This measure, called domain score, can either be used in a meta-analysis or as a filter to reduce the set of input motifs. Using *MASSIF* improves the PR-AUC of the tools CentriMo and PASTAA significantly. For shorter sequences, the best results are observed for the variant where we apply PASTAA and CentriMo on the reduced motif set, and combine the results with Fisher’s method and for longer sequences *M_C_* is the best variant.

Since we use a similarity measure based on PFMs, *MASSIFs’* domain score can be applied to improve the performance of any tool that links motifs to TFs if it is based on motifs in a matrix representation. More complex representations of DNA motifs could also be included if a suitable similarity measure exists. Even if a tool does not provide a *P*-value as part of its analysis, our domain score can be used as a filter to improve results. The only additional information *MASSIF* requires in comparison to most of the tools that link motifs to TFs based on TF-associated sequences, is the DBD of the TF of interest. It is practicable to determine the DBD of a TF even if the DBD is not listed in a protein database like UniProt by predicting it using protein domain profiles (El-Gebali *et al.*, 2019). However, in case this is not possible or if the domain type is not included in our current DBD collection, our current method cannot be used to improve the result.

To demonstrate our approach in a realistic setting, we applied it to 102 human sequence sets resulting from ChIP-seq experiments. The TFs and the TF-associated sequences considered had diverse characteristic. As a motif input set, we used either all motifs available in the motif database HOCOMOCO or *de novo* motifs for evaluation purposes. In practice, any motif set can be used, from another motif database, or experimentally derived or *de novo* motifs.

In our experiments, we investigated sequence sets with differing length, which illustrated that our approach produces stable results for all lengths. This is important, as peak length may vary with experiment quality and the used peak caller.

The advantage of using the domain scores in a meta-analysis is that no threshold has to be selected. However, using Fisher’s method might not be the optimal statistical test to combine the domain score with the *P*-values of tools that link motifs to TFs based on TF-associated sequences. The smallest possible *P*-value of the domain score using default parameters is $10^{-5}$, since the corresponding distribution is based on 100 000 random motifs. Compared to the smallest possible *P*-values of CentriMo and PASTAA, this *P*-value is rather big. Fisher’s method is more sensitive to smaller *P*-values (Heard and Rubin-Delanchy, 2018). Thus, depending on the resolution, this kind of approach may favor *P*-values from either CentriMo or PASTAA. On the other hand, if we use the domain score as a filter and combine the results of PASTAA and CentriMo, this bias is less problematic, as the *P*-values of both tools are in the same range. This could possibly explain why using the domain score as a filter achieves better PR-AUC than using the domain score within Fisher’s method. However, the drawback of the filter-based approach is the need to pick a *P*-value threshold. We selected a value between the two extremes by picking the *P*-value as 0.001, which may be further improved.

The idea to combine two or more tools, has been applied in the context of *de novo* motif discovery tools or methods that link motifs to TFs based on TF-associated sequences, e.g. MotifViz (Fu *et al.*, 2004), completeMOTIFs (Kuttippurathu *et al.*, 2011) or MEME-ChIP (Ma *et al.*, 2014). Nevertheless, none of these approaches combine the results of multiple tools in a statistical analysis.

Clearly, the observed results are also depending on the used tools, that link motifs to TFs based on TF-associated sequences. CentriMo, for instance uses flanking regions around the peaks to simulate a background distribution. Choosing too narrow peaks leads to worse results since the background is not represented reasonably, as we observed for the sequence length 100 bp and 300 bp. In contrast, PASTAA uses TRAP (Roider *et al.*, 2007) to estimate a TF binding affinity for all sites in each sequence. The longer the sequences, the more sites are incorporated, which may lead to a less accurate affinity computation. As we observed, longer sequences decrease the number of correctly linked motifs.

It is difficult to decide whether known motifs or *de novo* motifs should be used as input. While using known motifs is faster and leads to a more reliable results, as the used motifs are known, the downside is that no new motifs can be identified. Hence, if the motif of the studied TF is not similar to any already known motif, this might be a problem. A solution can be to use *de novo* motifs as input. However, for roughly 50% of the datasets, *GimmeMotifs* was not able to identify significant motifs and for one-third of the remaining datasets all motifs were excluded by the filtering. In addition, the list of identified *de novo* motifs can include motifs of the co-factors of the TF of interest as well as motifs based on repetitive sequences. Further, depending on the quality of the dataset and the characteristics of the motif of the TF of interest, the used *de novo* motif discovery algorithm might not be able to find the true motif.

An opportunity to improve the performance of *MASSIF* could be to refine the DBD collection such that large DBD families like ‘Homeo domain factors’ or ‘C2H2 zinc finger factors’ are split into multiple smaller ones. By using the domain score as a filter, we observed the tendency that for smaller DBD families a higher improvement is possible than for the larger ones (Fig. 4c). However, we decided against this additional splitting, because otherwise it could become difficult for the user to assign the TF of interest to the corresponding DBD.

In summary, we demonstrate that a commonly available and easy to access information of the TF, namely the DBD can be used as additional information to significantly improve the performance of tools that link motifs to TFs based on TF-associated sequences. *MASSIF* is freely available online at https://github.com/SchulzLab/MASSIF.

## Funding

This work was supported by the DZHK (German Centre for Cardiovascular Research, 81Z0200101) and the DFG Clusters of Excellence on Multimodal Computing and Interaction [EXC248] and Cardio-Pulmonary Institute (CPI) [EXC 2026].

*Conflict of Interest*: none declared.

## Supplementary Material

btz855_Supplementary_Data
