# Supplementary material for: Improved linking of motifs to their TFs using domain information
Source: Bioinformatics. 2019 Nov 19;36(6):1655–62. doi: 10.1093/bioinformatics/btz855 (PMC7703792; doi:10.1093/bioinformatics/btz855)
Supplement: btz855_Supplementary_Data [file btz855_supplementary_data.pdf]

Supplementary

Improved linking of motifs to their TFs using domain information

Nina Baumgarten, Florian Schmidt and Marcel H. Schulz

November 8, 2019

Supplementary Section S1. Used commands of CentriMo, PASTAA and GimmeMotifs

To ensure an easy reproducibility of our results, we list the CentriMo,PASTAA and GimmeMotifs commands below.

CentriMo

Step1: Convert the PFMs into meme format

```
CentriMo/scripts/transfac2meme transfac_file.txt
>PWMsInMemeFormat.txt
```

Step2: Call CentriMo for each ChIP-seq data set

```
CentriMo/src/centrimo --oc centrimo_TF  --ethresh
1000000000 fasta_dir/TF.fa PWMsInMemeFormat.txt
```

When using the domain score as a filter, the first step needs to be repeated for every sequence set, since the set of PFMs is differing. We downloaded the software from http://meme-suite.org/doc/download.html and used throughout the project meme-5.0.4.

PASTAA

Step1: Motifs represented as PFMs are converted into Position Score Energy Matrices (PSEMs)

```
./src/PSCM_to_PSEM transfac_file.txt >energy.txt
```

Step2: Use TRAP to determine the sum over all binding probabilities of a motif in a sequence

```
./src/TRAP energy.txt fasta_dir/TF.fa >affinity_TF.txt
```

Step3: Run PASTAA on each ChIP-seq data set

```
./src/PASTAA affinity_TF.txt biological_signal/
gene_list_TF.txt | sort -k2,2 -g >enrichment_TF.txt
```

PASTAA was downloaded from `http://trap.molgen.mpg.de/PASTAA/`.

GimmeMotifs

Step1: Run GimmeMotifs for each ChIP-seq data set

```
gimme motifs TF.bed -n GimmeMotifs_TF/ -g hg38
```

We installed GimmeMotifs via conda and used the version gimmemotifs-0.13.1 in this project.

Supplementary Section S2. Accession numbers

Supplementary Table S 1 shows for each TF ChIP-seq data set considered in the ENCODE data set the accession number. TODO: nochmal nachsehen

Supplementary Section S3. DBDs observed in the JASPAR database

Supplementary Table S 2 lists the 30 DBDs observed within the JASPAR database and the structural class according to the TFClass system.

Supplementary Section S4. Detailed information about the DBD collection

In the following for each DBD observed in the JASPAR database the clusters and the corresponding motifs per cluster are listed.

DBD 1 cluster 1: AHR::ARNT cluster 2: MAX::MYC cluster 3: TAL1::TCF3 cluster 4: HAND1::TCF3 cluster 5: GATA1::TAL1 cluster 6: ARNT::HIF1A cluster 7: NEUROD2 cluster 8: CLOCK cluster 9: ASCL2 cluster 10: TFE3 cluster 11: TWIST2 cluster 12: HES1 cluster 13: MYF6 cluster 14: FIGLA cluster 15: MSC cluster 16: TCF7 cluster 17: MITF cluster 18: NPAS2 cluster 19: OLIG3 cluster 20: TFEB cluster 21: ARNTL cluster 22: MNT cluster 23: TCFL5 cluster 24: BHLHA15 cluster 25: HEY2 cluster 26: NEUROG2 cluster 27: TFEC cluster 28: NEUROG1 cluster 29: TCF7L2 cluster 30: HES228 cluster 31: ID4 cluster 32: ATOH1 cluster 33: ARNT cluster 34: SPZ1 cluster 35: SREBF1(VAR.2) SREBF2(VAR.2) cluster 36: SREBF1 SREBF2 cluster

37: MYC MYCN cluster 38: USF2 USF1 cluster 39: NHLH1 MYOD1 TCF12 MYOG TCF21 TFAP4 cluster 40: TCF3 TCF4 cluster 41: MAX HEY1 MLX MLXIPL ID2 MLXIP BHLHE40 BHLHE41 HES7 HES5 cluster 42: OLIG2 BHLHE22 BHLHE23 OLIG1

DBD 2 cluster 1: DDIT3::CEBPA cluster 2: MAFG::NFE2L1 cluster 3: FOS::JUN cluster 4: BATF::JUN cluster 5: CREB1 cluster 6: ATF4 cluster 7: CREB3L2 cluster 8: ATF1 cluster 9: CEBPG cluster 10: CEBPD cluster 11: MAFG cluster 12: HLF cluster 13: CEBPA cluster 14: NFIL3 cluster 15: MAFF cluster 16: NRL cluster 17: MAFB cluster 18: CEBPB cluster 19: DBP cluster 20: ATF3 cluster 21: CEBPE cluster 22: NRF1 cluster 23: MAFK cluster 24: CREM cluster 25: NFE2L2 MAF::NFE2 BACH1::MAFK cluster 26: CREB3L1 CREB3 XBP1 cluster 27: CREB5 ATF7 JDP2(VAR.2) BATF3 JUND(VAR.2) JUN cluster 28: JDP2 FOS JUN(VAR.2) FOSL2 JUNB FOSL1 JUND NFE2

DBD 3 cluster 1: PPARG::RXRA cluster 2: RARA::RXRA cluster 3: NR1H3::RXRA cluster 4: ESR1 cluster 5: NR2F1 cluster 6: ESR2 cluster 7: NR5A2 cluster 8: HNF4G cluster 9: RARG(VAR.2) cluster 10: NR4A2 cluster 11: RORA cluster 12: RORA(VAR.2) cluster 13: ESRRG cluster 14: NR2E3 cluster 15: NR2E1 cluster 16: PPARG cluster 17: NR2F6(VAR.2) RARA RARG RARB cluster 18: RARA(VAR.2) RARB(VAR.2) cluster 19: AR NR3C2 NR3C1 cluster 20: RXRA::VDR VDR cluster 21: ESRRB ESRRA cluster 22: NR2C2 NR2F6 HNF4A NR1H2::RXRA RXRA RXRB RXRG

DBD 4 cluster 1: NFIC::TLX1 cluster 2: HOXA5 cluster 3: ALX3 cluster 4: HOXA2 cluster 5: NKX6-1 cluster 6: MEOX2 cluster 7: PROX1 cluster 8: PRRX2 cluster 9: GSX1 cluster 10: RHOXF1 cluster 11: SIX3 cluster 12: HOXB2 cluster 13: UNCX cluster 14: EN2 cluster 15: NKX2-5 cluster 16: NKX2-5(VAR.2) cluster 17: LMX1A cluster 18: EMX1 cluster 19: LHX3 cluster 20: DLX1 cluster 21: LHX8 cluster 22: PBX1 cluster 23: ZEB1 cluster 24: LMX1B cluster 25: HMBOX1 cluster 26: LHX4 cluster 27: HOXD8 cluster 28: NOTO cluster 29: MIXL1 cluster 30: HOXB3 cluster 31: GSX2 cluster 32: ISL2 cluster 33: VAX2 cluster 34: MNX1 cluster 35: VAX1 cluster 36: BARHL2 cluster 37: BARHL1 cluster 38: NKX6-2 cluster 39: HNF1B HNF1A cluster 40: HMX1 HMX2 HMX3 cluster 41: DUX4 DUXA cluster 42: HOXC9 HOXA9 cluster 43: ONECUT2 ONECUT3 ONECUT1 CUX2 CUX1 cluster 44: POU4F1 POU4F3 POU4F2 cluster 45: HOXC10 HOXC13 HOXA11 HOXD11 HOXC11 HOXC12 HOXD12 cluster 46: PROP1 PHOX2A PHOX2B cluster 47: POU5F1B POU3F1 POU2F1 POU2F3 POU2F2 POU3F4 POU3F3 POU3F2 POU1F1 cluster 48: HOXD3 HOXB5 cluster 49: NKX3-2 NKX3-1 NKX2-3 NKX2-8 cluster 50: VSX2 VSX1 cluster 51: EVX2 EVX1 cluster 52: GSC2 PITX1 PITX3 GSC OTX1 OTX2 DMBX1 CRX cluster 53: BSX BARX1 VENTX cluster 54: MEIS1 TGIF2 PKNOX2 TGIF1 PKNOX1 MEIS2 MEIS3 cluster 55: LBX1 POU6F2 MEOX1 cluster 56: DLX4 DLX3 RAX2 LHX9 SHOX ESX1 LBX2 PRRX1 DLX6 DLX2 PDX1 MSX1 POU6F1 NOBOX LHX6 ARX ALX4 ALX1 LHX2 SHOX2 EN1 EMX2 RAX MSX2 ISX HESX1 GBX2 GBX1 cluster 57: HOXD9 HOXA10 CDX1 HOXA13 CDX2 HOXD13 HOXB13

DBD 5 cluster 1: POU5F1::SOX2 cluster 2: LEF1 cluster 3: SOX9 cluster 4: SRY cluster 5: SOX1 cluster 6: SOX5 cluster 7: SOX6 cluster 8: SOX3 cluster 9: SOX17 cluster 10: SOX2 cluster 11: SOX10 cluster 12: SOX8 cluster 13: SOX21 SOX11 SOX4

DBD 6 cluster 1: EWSR1::FLI1 cluster 2: FEV cluster 3: ELF5 cluster 4: ERF cluster 5: ETV5 cluster 6: MYB cluster 7: MYBL2 cluster 8: ETV6 cluster 9: SPIB cluster 10: HLTF cluster 11: SPDEF cluster 12: ERG cluster 13: ETV4 cluster 14: SPIC cluster 15: ETS1 cluster 16: ETV3 cluster 17: ETV1 cluster 18: SPI1 cluster 19: MYBL1 cluster 20: ELK3 cluster 21: IRF1 IRF7 IRF2 IRF9 IRF8 cluster 22: EHF ELF4 ELF3 ELF1 ELK4 GABPA cluster 23: ELK1 FLI1 ETV2

DBD 7 cluster 1: SMAD2::SMAD3::SMAD4 cluster 2: NFIC cluster 3: SMAD3 cluster 4: NFIA NFIX

DBD 8 cluster 1: STAT1::STAT2 cluster 2: STAT5A::STAT5B cluster 3: STAT6 cluster 4: STAT3 cluster 5: STAT4 STAT1

DBD 9 cluster 1: GATA4 cluster 2: GATA5 cluster 3: GATA2 cluster 4: GATA3 GATA1

DBD 10 cluster 1: NFAT5 cluster 2: EBF1 cluster 3: NFATC3 cluster 4: REL cluster 5: RELA cluster 6: NFATC2 cluster 7: NFATC1 cluster 8: NFKB1 NFKB2

DBD 11 cluster 1: HSF4 cluster 2: HSF1 HSF2

DBD 12 cluster 1: TP73 cluster 2: TP63 TP53

DBD 13 cluster 1: CTCF cluster 2: GFI1 cluster 3: GLI2 cluster 4: ZNF740 cluster 5: ZNF423 cluster 6: SP8 cluster 7: ZBTB18 cluster 8: MECOM cluster 9: GFI1B cluster 10: REST cluster 11: ZBED1 cluster 12: ZNF263 cluster 13: HIC2 cluster 14: PLAG1 cluster 15: ZNF354C cluster 16: ZFX cluster 17: YY2 cluster 18: PRDM1 cluster 19: RREB1 cluster 20: HIC1 cluster 21: ZBTB33 cluster 22: YY1 cluster 23: HINFP cluster 24: INSM1 cluster 25: MTF1 cluster 26: SNAI2 cluster 27: MZF1 cluster 28: MZF1(VAR.2) cluster 29: SCRT1 SCRT2 cluster 30: GLIS2 GLIS3 GLIS1 ZIC4 ZIC1 ZIC3 cluster 31: BCL6 BCL6B cluster 32: ZNF410 ZNF143 cluster 33: ZBTB7C ZBTB7B ZBTB7A cluster 34: EGR1 EGR4 EGR2 EGR3 cluster 35: SP3 KLF5 SP2 SP1 KLF16 KLF12 KLF14 SP4 KLF13 KLF1 KLF4

DBD 14 cluster 1: THAP1

DBD 15 cluster 1: FOXO4 cluster 2: FOXD3 cluster 3: FOXD2 cluster 4: FOXQ1 cluster 5: FOXH1 cluster 6: FOXL1 cluster 7: FOXB1 cluster 8: FOXC1 cluster 9: FOXO3 cluster 10: FOXI1 cluster 11: FOXC2 cluster 12: FOXP3 cluster 13: FOXD1 cluster 14: FOXP1 cluster 15: E2F6 E2F4 E2F8 E2F7 cluster 16: RFX1 RFX4 RFX3 RFX5 RFX2 cluster 17: FOXO1 FOXP2 cluster 18: E2F1 E2F3 E2F2 cluster 19: FOXA1 FOXA2 cluster 20: FOXJ2 FOXO6 FOXF2 FOXG1 cluster 21: FOXK1 FOXJ3

DBD 16 cluster 1: TBX2 cluster 2: TBR1 cluster 3: TBX21 cluster 4: TBX20 cluster 5: MGA cluster 6: TBX5 cluster 7: TBX4 cluster 8: EOMES cluster 9: TBX19 T cluster 10: TBX15 TBX1

DBD 17 cluster 1: MEF2C cluster 2: SRF cluster 3: MEF2A cluster 4: MEF2D MEF2B

DBD 18 cluster 1: DMRT3
DBD 19 cluster 1: ARID5A cluster 2: ARID3A ARID3B
DBD 20 cluster 1: NFYB NFYA
DBD 21 cluster 1: PAX4 cluster 2: PAX6 cluster 3: PAX5 cluster 4: PAX2 cluster 5: PAX9 PAX1 cluster 6: PAX3 PAX7
DBD 22 cluster 1: TFAP2B cluster 2: TFAP2B(VAR.2) cluster 3: TFAP2A cluster 4: TFAP2A(VAR.2) cluster 5: TFAP2A(VAR.3) cluster 6: TFAP2C cluster 7: TFAP2C(VAR.2) cluster 8: TFAP2B(VAR.3) TFAP2C(VAR.3)
DBD 23 cluster 1: RUNX1 cluster 2: RUNX3 RUNX2
DBD 24 cluster 1: TEAD1 cluster 2: TEAD4 cluster 3: TEAD3 cluster 4: TEF
DBD 25 cluster 1: GCM1 GCM2
DBD 26 cluster 1: TBP
DBD 27 cluster 1: TFCP2 GRHL1
DBD 28 cluster 1: GMEB1 GMEB2
DBD 29 cluster 1: CENPB
DBD 30 cluster 1: LIN54

Supplementary Section S5. Using *de novo* motifs as input for the TF ChIP-seq data sets

In Figure 1 some example of linked *de novo* motifs in comparison to the true motifs are shown.

Supplementary Section S6. Generation of random PFMs

To determine the domain score distribution we randomly sampled motifs. The length of the random motifs is chosen between 6 and 21, since we observed that most of the motifs of the JASPAR database lie also in that range (see Figure 2a). In addition, we noticed that extreme short and extreme long motifs appear rather rare. To mimic that behavior we used a normal distribution with mean 12 and variation 0.3 from which we pick randomly numbers in the range of 6 to 21. For each entry of the matrix we picked a number based on a uniform distribution between 1 and 100. We add up the number of each column and divide the entries per column by this number. For each random motif we calculated the overall entropy and if it was equal to or higher -0.6, we kept the motif. We picked that average entropy cutoff based on the average entropy of the JASPAR motifs (see also Figure 2b).

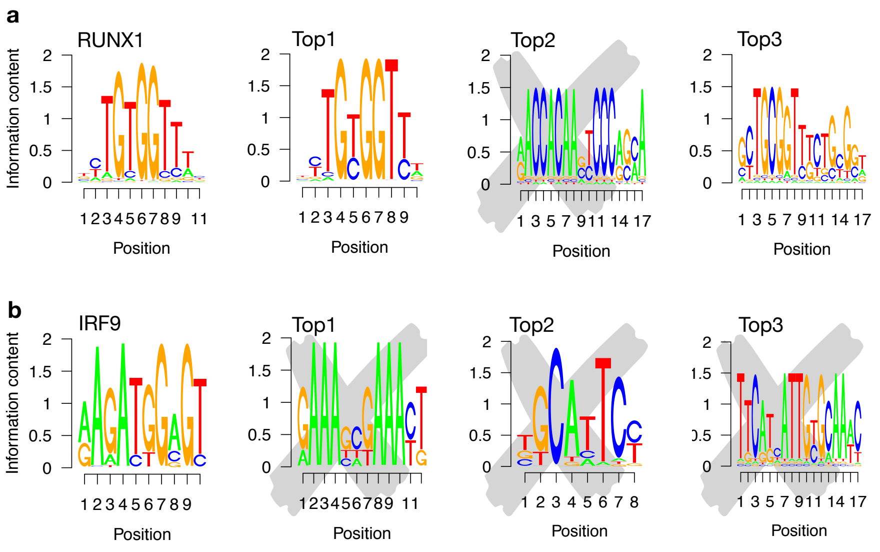

Figure 1: Examples of *de novo* identified motifs in comparison to the true ones. The first motif per row is the known motif of the current TF ChIP-seq data set, the following motifs are *de novo* motifs sorted according to their similarity to the true motif. A grey cross marks a motif that is eliminated throughout the filtering step. a) shows an example where the linked motif from *GimmeMotifs* is similar to the true one as well as the case where the filtering excludes a *de novo* motif that fit not properly to the true one. b) outlines an example, where the filtering eliminates all *de novo* motifs. In such cases MASSIF is not able to make a prediction.

Supplementary Section S7. Motifs incorrectly linked to TFs

In the following we list all TFs with incorrectly linked motifs separately for each variant.

TF	P	C	CP	MCP	M_P	M_C	M_{CP}
ARID3A	×	×	×	×			
ARNT	×	×	×	×	×	×	×
ATF1	×	×	×	×	×	×	×
ATF2	×	×	×	×	×	×	×
ATF3	×	×	×	×	×	×	×
ATF7	×	×	×	×	×	×	×
BACH1							
BHLHE40							
CEBPB	×				×		
CEBPG							
CREB3	×	×	×	×	×		
CREB3L1	×	×					
CREM							
CTCF							

CTCFL	×				×		
CUX1							
E2F1							
E2F4	×	×	×	×		×	×
E2F5	×	×	×	×			
E2F6	×	×	×	×		×	
E2F7							
EGR1		×					
ELF1							
ELF4	×						
ELK1							
ESRRA							
ETS1	×	×	×		×		
ETS2	×	×	×		×	×	
ETV1							
ETV6	×	×	×	×	×		×
FOSL1	×						
FOXA1							
FOXJ2	×	×	×	×	×	×	×
GABPA							
GATA1	×		×				
GATA2	×		×				
HES1	×	×	×	×	×	×	×
HINFP	×		×	×	×		×
HMBOX1	×	×	×	×	×	×	×
IKZF1	×	×	×	×	×	×	×
IRF1	×	×	×			×	
IRF2							
IRF9	×	×	×	×	×	×	×
JUN	×	×	×	×	×	×	×
JUNB	×						
JUND							
KLF1	×						
KLF13							
KLF16	×	×	×	×	×	×	×
LEF1	×	×	×	×	×	×	×
MAFF							
MAFG							
MAFK							
MAX							
MEF2A	×	×	×				
MEF2D	×	×	×				
MGA	×	×	×	×			
MITF							
MNT							
MYBL2	×	×	×	×	×	×	×

MYC							
NFE2							
NFIC							
NFYA							
NFYB	×						
NR2C1	×		×				
NR2C2	×						
NR2F1							
NR2F6	×		×				
NR4A1	×	×	×	×	×	×	×
NRF1							
PBX2	×	×	×	×	×	×	×
PKNOX1	×	×	×	×	×	×	×
RELA	×	×	×	×	×	×	×
REST		×			×		
RFX1	×						
RFX5	×	×	×	×	×		×
RUNX1							
SMAD2	×	×	×	×	×	×	×
SOX6	×	×	×	×			
SP1	×				×		
SREBF1		×	×	×		×	×
STAT1	×	×	×	×	×		×
STAT2	×	×	×	×	×		×
TAL1	×						
TBP	×	×	×				×
TCF12	×	×	×		×	×	×
TCF7		×				×	
TCF7L2	×						
TEAD4	×	×	×	×	×	×	×
TFDP1		×	×	×			
THAP1	×	×	×	×			
USF1							
USF2							
YY1							
ZBED1	×	×	×	×	×	×	×
ZBTB33	×	×	×			×	×
ZBTB7A		×					
ZFX							
ZNF143		×		×	×	×	×
ZNF263	×	×	×		×	×	×
ZNF740	×				×	×	×

Supplementary Table S 3: TFs with incorrectly linked motifs for all variants. The cross indicates TFs for which a incorrect motif was linked.

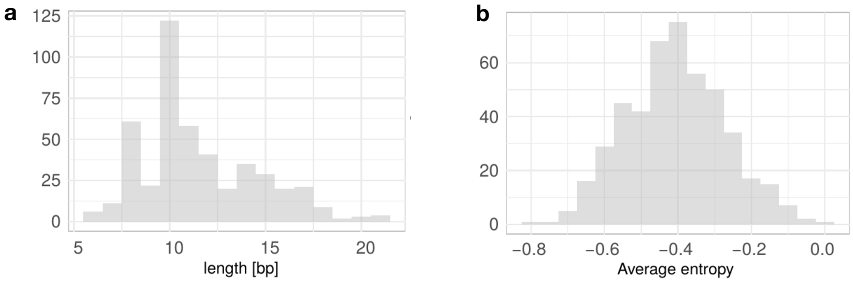

Figure 2: Bar plots represent detailed information of the length and the entropy of the JASPAR motifs. The y-axis outlines the number of motifs per category. a) separates the JASPAR motifs according to their observed length (x-axis) and b) according to the observed average entropy (x-axis)

TF	accession number	TF	accssion number
ZFX	ENCFF572OWY.bed	JUN	ENCFF394CEC.bed
MAFK	ENCFF002CXC.bed	MAFF	ENCFF002CXB.bed
MAFG	ENCFF393CCU.bed	ZNF143	ENCFF002CYR.bed
BHLHE40	ENCFF002CVQ.bed	ZNF263	ENCFF002CYS.bed
RELA	ENCFF931SVP.bed	NFIC	ENCFF092TVM.bed
SP1	ENCFF452LDK.bed	CREB3	ENCFF606EUI.bed
NR2C2	ENCFF789AXP.bed	NR2C1	ENCFF664ZGR.bed
GATA2	ENCFF002CMA.bed	GATA1	ENCFF632NQI.bed
RFX5	ENCFF002CXV.bed	ARNT	ENCFF447FIO.bed
RFX1	ENCFF010UHD.bed	TCF7L2	ENCFF556FYF.bed
FOXA1	ENCFF765NAN.bed	RUNX1	ENCFF545WXN.bed
TCF12	ENCFF912LXU.bed	CTCF	ENCFF002CLS.bed
HINFP	ENCFF558VAL.bed	TCF7	ENCFF512IAI.bed
ESRRA	ENCFF592GWM.bed	BACH1	ENCFF365WQR.bed
EGR1	ENCFF558JBX.bed	MNT	ENCFF459DYU.bed
ELK1	ENCFF002CWO.bed	NFE2	ENCFF312XHI.bed
ZBTB33	ENCFF002CMY.bed	FOXJ2	ENCFF175IUD.bed
CUX1	ENCFF556HMX.bed	E2F7	ENCFF013EHI.bed
PKNOX1	ENCFF062VBB.bed	ETV1	ENCFF804KSB.bed
IRF2	ENCFF886EVL.bed	MGA	ENCFF525MPI.bed
ETV6	ENCFF660VPM.bed	TBP	ENCFF002CYK.bed
IRF9	ENCFF863WCN.bed	MEF2A	ENCFF002CMD.bed
CREM	ENCFF021XJN.bed	MEF2D	ENCFF257RYT.bed
HES1	ENCFF010OOE.bed	TFDP1	ENCFF600MGE.bed
YY1	ENCFF002CMX.bed	SMAD2	ENCFF186MFI.bed
IKZF1	ENCFF994OQH.bed	STAT2	ENCFF002CYG.bed
STAT1	ENCFF002CYB.bed	ZNF740	ENCFF301FKQ.bed
NR2F6	ENCFF712AXK.bed	NR2F1	ENCFF363IQN.bed
USF2	ENCFF002CYP.bed	USF1	ENCFF002CMV.bed
FOSL1	ENCFF002CLY.bed	ZBED1	ENCFF388TYU.bed
TAL1	ENCFF002CYH.bed	E2F6	ENCFF002CLU.bed
IRF1	ENCFF002CWW.bed	MAX	ENCFF002CXD.bed
LEF1	ENCFF043YZF.bed	MYC	ENCFF002CWD.bed
NFYB	ENCFF002CXJ.bed	SREBF1	ENCFF777MYW.bed
GABPA	ENCFF002CLZ.bed	NRF1	ENCFF782YFS.bed
JUNB	ENCFF739XTO.bed	JUND	ENCFF002CWZ.bed
ELF1	ENCFF617ZLL.bed	NFYA	ENCFF002CXI.bed
MITF	ENCFF071NYD.bed	MYBL2	ENCFF905KOD.bed
SOX6	ENCFF431STY.bed	ELF4	ENCFF539SXG.bed
TEAD4	ENCFF002CMT.bed	KLF13	ENCFF381GEK.bed
CEBPB	ENCFF002CVV.bed	CEBPG	ENCFF086CSF.bed
KLF16	ENCFF844HBQ.bed	CTCFL	ENCFF002CLT.bed
ETS2	ENCFF772HOY.bed	ETS1	ENCFF002CLX.bed
KLF1	ENCFF287GDT.bed	ZBTB7A	ENCFF002CMZ.bed
NR4A1	ENCFF859NPS.bed	REST	ENCFF002CMF.bed
PBX2	ENCFF269EMM.bed	ARID3A	ENCFF002CVL.bed
THAP1	ENCFF002CMU.bed	E2F5	ENCFF468VJV.bed
E2F4	ENCFF002CWM.bed	E2F1	ENCFF445VTT.bed
HMBOX1	ENCFF718DFX.bed	CREB3L1	ENCFF566HGU.bed
ATF7	ENCFF371SJR.bed	ATF1	ENCFF030HWZ.bed
ATF3	ENCFF002CLN.bed	ATF2	ENCFF803FHN.bed

Supplementary Table S 1: Accession numbers of the downloaded ENCODE TF ChIP-seq data set.

DBD number	DBD	# clusters
DBD 1	Basic helix-loop-helix factors (bHLH)	42
DBD 2	Basic leucine zipper factors (bZIP)	28
DBD 3	Nuclear receptors with C4 zinc fingers	22
DBD 4	Homeo domain factors	57
DBD 5	High-mobility group (HMG) domain factors	13
DBD 6	Tryptophan cluster factors	23
DBD 7	SMAD/NF-1 DNA-binding domain factors	4
DBD 8	STAT domain factors	5
DBD 9	Other C4 zinc finger-type factors	4
DBD 10	Rel homology region (RHR) factors	8
DBD 11	Heat shock factors	2
DBD 12	p53 domain factors	2
DBD 13	C2H2 zinc finger factors	35
DBD 14	C2CH THAP-type zinc finger factors	1
DBD 15	Fork head / winged helix factors	21
DBD 16	T-Box factors	10
DBD 17	MADS box factors	4
DBD 18	DM-type intertwined zinc finger factors	1
DBD 19	ARID domain factors	2
DBD 20	Heteromeric CCAAT-binding factors	1
DBD 21	Paired box factors	6
DBD 22	Basic helix-span-helix factors (bHSH)	8
DBD 23	Runt domain factors	2
DBD 24	TEA domain factors	4
DBD 25	GCM domain factors	1
DBD 26	TATA-binding proteins	1
DBD 27	Grainyhead domain factors	1
DBD 28	SAND domain factors	1
DBD 29	Psq-type HTH domain	1
DBD 30	CRC domain	1

Supplementary Table S 2: DNA-binding domains of the TFs within the JASPAR database.
